# FSHR Trans-Activation and Oligomerization

**DOI:** 10.3389/fendo.2018.00760

**Published:** 2018-12-13

**Authors:** Kamila Szymańska, Joanna Kałafut, Alicja Przybyszewska, Beata Paziewska, Grzegorz Adamczuk, Michał Kiełbus, Adolfo Rivero-Müller

**Affiliations:** ^1^Department of Biochemistry and Molecular Biology, Medical University of Lublin, Lublin, Poland; ^2^Independent Medical Biology Unit, Medical University of Lublin, Lublin, Poland; ^3^Cell Biology, Biosciences, Faculty of Science and Engineering, Åbo Akademi University, Turku, Finland

**Keywords:** follicle-stimulating hormone (FSH), follicle-stimulating hormone receptor (FSHR), G protein-coupled receptor (GPCR), transactivation, biased signaling, oligomerization, homodimers, heterodimers

## Abstract

Follicle stimulating hormone (FSH) plays a key role in human reproduction through, among others, induction of spermatogenesis in men and production of estrogen in women. The function FSH is performed upon binding to its cognate receptor—follicle-stimulating hormone receptor (FSHR) expressed on the surface of target cells (granulosa and Sertoli cells). FSHR belongs to the family of G protein-coupled receptors (GPCRs), a family of receptors distinguished by the presence of various signaling pathway activation as well as formation of cross-talking aggregates. Until recently, it was claimed that the FSHR occurred naturally as a monomer, however, the crystal structure as well as experimental evidence have shown that FSHR both self-associates and forms heterodimers with the luteinizing hormone/chorionic gonadotropin receptor—LHCGR. The tremendous gain of knowledge is also visible on the subject of receptor activation. It was once thought that activation occurs only as a result of ligand binding to a particular receptor, however there is mounting evidence of trans-activation as well as biased signaling between GPCRs. Herein, we describe the mechanisms of aforementioned phenomena as well as briefly describe important experiments that contributed to their better understanding.

## Introduction

Gonadotrophin hormones, which include luteinizing hormone (LH), follicle stimulating hormone (FSH) and human chorionic gonadotropin (hCG), perform a number of functions pivotal for the process of sexual development and reproduction as well as for the fetal development in the case of the latter hormone. Alongside the thyroid-stimulating hormone (TSH) they comprise a glycoprotein hormone family. LH, FSH, and TSH are synthesized and secreted by the cells of the anterior pituitary gland (gonadotrophs), while hCG is produced by placental syncytiotrophoblasts.

The action of these hormones is achieved by the presence of receptors belonging to the class A of G protein-coupled receptors (GPCRs)—FSH constitutes ligand for FSHR, TSH for TSHR, whereas LH and hCG for their common LHCGR (1). GPCRs constitute the largest protein superfamily and the most various group of all membrane receptors in human genome, and they transmit a broad spectrum of extracellular signals in cell physiology and homeostasis (2, 3). Receptors belonging to the GPCRs are distinguished by the presence of transmembrane domain built of seven α-helices and coupling with G proteins responsible for the signal transduction following the activation of receptor with corresponding ligand. Due to their structure, one could define three areas of the protein: the extracellular domain (*N*-terminus and three extracellular loops), transmembrane domain, and the intracellular domain (three intracellular loops as well as the *C*-terminus) (4). Glycoprotein receptors differ from other class A GPCRs due to the presence of large *N*-terminal domain (exodomain) within which the ligand-binding site is located (5, 6). Their exodomain is composed of nine subdomains containing leucine-rich repeats (LRR) as well as cysteine-rich subdomains located at the *C*- and *N*-terminus of this domain, respectively (7).

Binding of LH/hCG, TSH or FSH to their cognate receptors results in activation of intracellular signaling pathways which in turn leads to the stimulation of cell growth, differentiation, and proliferation. In the case of FSHR and LHCGR, the cyclic AMP (cAMP) pathway plays a major role in intracellular signaling and it involves the coupling of receptor with G_α*s*_ protein that is responsible for activation of adenylyl cyclase and thereby an increased level of cAMP. Aside from the cAMP pathway, other signaling pathways can be distinguished herein, including the extracellular regulated kinases (ERKs), phosphatidylinositol-3 kinase (PI3K), protein kinase B (PKB), p38 mitogen-activated protein kinases (MAPKs), and protein kinase C (PKC) pathways (8, 9). Although not all of them are solely G protein-dependent, such as the ERK pathway which can also be activated via β-arrestins (10).

The major expression sites of FSHR, posing the subject of this review article, constitute Sertoli cells in the testis and granulosa cells in the ovary (11). Extragonadal FSHR expression has been detected in a variety of other cell types and tissues including osteoclasts (12, 13), human umbilical vein endothelial cells (14), monocytes (15, 16), female reproductive tract (17), and liver (18), although the functional and physiological significance of this is debatable (19). FSH stimulates estrogen production by granulosa cells, the growth and maturation of ovarian follicles as well as it regulates the ovulatory cycle, whereas in males it induces the secretion of androgen-binding protein, stimulates Sertoli cells and thereby the spermatogenesis process (5).

In the last one and a half decades, GPCRs have been found to form functional oligomers composed of two or more receptors (20). Furthermore, these protomers are built of either one type or different types of GPCRs, thus they can form homo- and/or heterodimers. The significance of GPCR oligomerization for proper cellular functioning consists in regulation of intracellular signaling via diversification and/or modulation of the signal, as well as during biosynthesis and desensitization. Presumably, monomers and oligomers remain in equilibrium, thus enabling the control of ligand action and intracellular signaling in response to ligand binding (21).

## GPCRs Oligomerization

**Figure 1:**
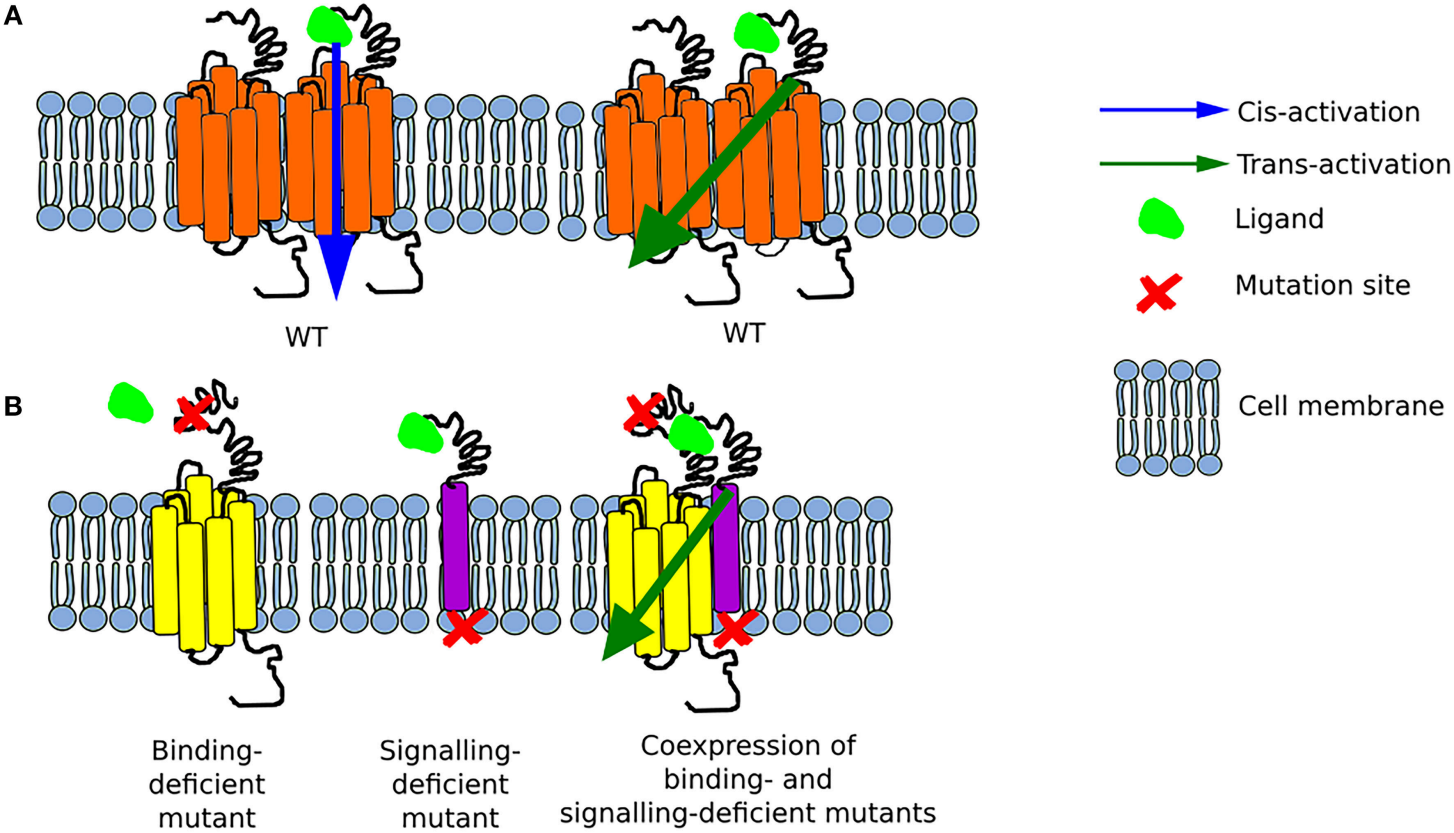
Schematic representation of possible options for GPCR activation **(A)** The function of the wild-type (WT) receptor is possible due to either cis-activation or trans-activation. **(B)** Two mutant receptors, a binding-deficient and a signaling-deficient are unable to function separately. Nonetheless, when they are co-expressed, they interact together and form a functional complex as a result of transactivation.

Oligomerization is a term used to describe the GPCR complexes composed of two (dimers), three (trimers) or higher number of protomers. Heretofore, a number of reports have been published about the formation of oligomers by receptors belonging to different classes of the GPCR family. Among the oligomers, we can distinguish homomers created by receptors of the same type, as well as heteromers composed of various closely related types of GPCRs (21). GPCRs must fulfill certain conditions to be classified as heteromers such as colocalization and physical interaction between protomers (Figure 1A). Additionally, they should be distinguished by acquisition of new properties, absence of monomers, or the loss of the characteristics typical for single protomers (22).

The first mention of presumable dimerization by GPCRs dates from the 1980s and it comes from the work of Birdsall (23). This conclusion was derived from experimental observations carried out by another research group several years earlier, where decreased binding affinity of β2 adrenergic receptor (β2AR) in the presence of GTP was noticed. Further treatment with methacholine, being the agonist for muscarinic cholinergic receptor, restored the binding affinity of β2AR, suggesting that the ligand binding by one receptor may affect the binding affinity of other receptor (24).

The first tangible evidence confirming the existence of oligomers among members of GPCRs family concerned mainly the C class of GPCRs, as several of these receptors are *forced oligomers*—they need to form oligomers to function (25). Co-immunoprecipitation studies performed by Romano et al. showed that metabotropic glutamate receptors (mGluRs) form dimers through the presence of one or more intermolecular disulphide bridges within the extracellular domain (26, 27). Yet, later they discovered that covalent dimerization is not a prerequisite for ligand binding but mGluRs form a non-covalent dimers in the absence of disulphide bridges (28). Simultaneously, the group led by Julia H. White studied the metabotropic γ-aminobutyric acid receptor, which was found to heterodimerize by two polypeptide chains within the carboxyl terminus, and its heterodimerization is required for its proper membrane expression as well as ligand binding affinity (29). Methodological developments have made possible to study oligomerization of GPCRs in more detail (see section *Methods to investigate the oligomerization of FSHR* for a better description of methods). For example, bioluminescence resonance energy transfer (BRET) was used by the group of Ali Salahpour for studying the homodimerization of β2AR. The researchers created β2AR mutants either carrying an endoplasmic reticulum (ER)-retention signal or lacking the ER-export motif as well as mutants with disturbed putative dimerization motif. All of the aforementioned mutant receptors formed dimers with the WT receptors, thus they inhibited the transport of WT β2AR to the cell membrane (30).

The abovementioned examples as well as many other dimer-forming C class of GPCRs provided evidence that made possible to ascertain that receptor dimerization is of vital importance for receptor functionality. Moreover, they have provided evidence for the existence of both homomerization and heteromerization at multiple levels in their live cycles. Homomerization of β2AR constitute an example of dimerization at the protein synthesis level. Alteration of one protomer results in inhibition of dimer trafficking, thus it prevents the expression of abnormal receptor on the cell membrane. Hence, dimerization seems to occasionally be a quality control mechanism during biosynthesis (31). Di/oligomerization may also occur during the next stages of receptor biosynthesis, such as protein maturation at Golgi apparatus as well as following ligand binding and activation of mature receptor on the plasma membrane (32).

Oligomer formation involves different regions of GPCRs with the special contribution of transmembrane domains and extracellular domains among receptors with large exodomains (31). Di- or oligomerization divided opinions: those who claimed there were no di/oligomers (seen as biochemical artifacts), those saying that di/oligomers existed but had no effect, and those that professed that only di/oligomers were functional. As often in biological systems, there was no single answer, instead more than 15 years of active research provided evidence that while some GPCRs work as monomers (*cis*-activation), others work in congregates, and others do both. What was less expected was the number of combinations these receptors can create, in particular at hetero-dimers/hetero-oligomers rendezvous. These aggregates often result in *allosteric modulation*—where the activation of one receptor can modify the affinity or responses of the second receptor to its ligand; or in the activation of (an) unliganded receptor(s) by the activation of another (or several) neighboring receptor(s)—this latter sequential signal activation is often called *receptor trans-activation*.

As many other GPCRs, the glycoprotein hormone receptors (LHCGR, TSHR and FSHR) have also been found to self-associate as well as to form heterodimers with other GPCRs (32–37). Moreover, the glycoprotein hormone receptors are also able to trans-activate, often in what seems to be another example of the regulatory mechanism for intracellular signaling via signal selection (38).

## GPCRs Trans-Activation

The first report on GPCR trans-activation was published by Daub et al. in (39), where they found that the epidermal growth factor receptor (EGFR), not a GPCR, became tyrosine-phosphorylated when rat fibroblasts were stimulated with GPCR agonists such as thrombin, endothelin-1 and lysoposphatic acid. These results provided evidence that the activation of a GPCR may result in the activation of another receptor, not necessarily another member of GPCR family (39).

It is worth mentioning that initially only *cis*-activation of GPCRs was postulated, meaning the activation of a single receptor (monomer) upon ligand binding (38). The phenomenon of trans-activation on the other hand, involves the interaction of two receptors either of the same type or different type. The earliest reports on GPCR trans-activation found that the ligand to one GPCR, e.g., muscarinic receptor, could alter, by binding to its own receptor, the affinity of a neighboring receptor e.g., β2AR for its own ligand (24). With the advent of molecular biology, the search for interacting GPCRs began, with unexpectedly large numbers of receptors found in dimers or higher order oligomers (20). The next problems to solve were to understand how these interactions affect the receptors, how to map the interacting domains between receptors, and whether oligomerization equals trans-activation (38).

Trans-activation of GPCRs has then been further elucidated by many elegant experiments using chimeric or mutant receptors, where only the presence of two complementary receptors would trigger downstream signaling (40). Large part of the interactions between heterodimeric receptors have been mapped to their transmembrane helices and *C*-terminal tails (29, 31).

### FSHR Trans-activation

Unlike other class A GPCRs, the glycoprotein hormone receptors (TSHR, LHCGR, and FSHR) have a very large extracellular *N*-terminal that is responsible for binding their respective hormones (41, 42). Such large extracellular binding arm could potentially reach another uncoupled receptor in a close proximity. In an attempt to test its feasibility, the group of Tae Ji generated a series of chimeric LHCGRs (37), and later on FSHRs (36), where the large *N*-terminal was fused to a membrane protein while a binding-deficient receptor was used as acceptor to transmit downstream signaling. The first set of mutants involved changes in the LRR of the LHCGR (or LHR as in some cases the mouse or rat LHR was used) as non-binding mutants, yet these still possess the intact transmembrane and intracellular domains to convey signaling. The first LHR signal-deficient mutant was created by substitution of lysine to arginine at position 605 of the polypeptide chain (initially referred as K578R due to old nomenclature). The co-expression of these two mutants in HEK293 cells resulted in binding of hCG, by the signal-deficient mutant, with the same affinity as in the case of the LHR WT, and a concomitant cAMP generation of about 30% of that of WT LHR via trans-activation of the binding-deficient mutant (37, 43).

Two years later, the same group of researchers created several FSHR constructs—some binding-deficient and the other signal-deficient. For the latter, the exodomain of FSHR (exoFSHR) was fused with either the glycosyl phosphatidylinositol (exoGPI) or the transmembrane domain of CD8 receptor (exoCD) (Figure 1B). Afterwards, stable lines expressing the signal-deficient FSHR mutants were transiently transfected with one of the plasmids encoding the exo-FSHR. The results revealed that a binding-deficient receptor could be trans-activated by signaling-deficient receptors. Unexpectedly, exoFSHR-GPI was able to activate the binding-deficient mutants FSHR^P24A^, FSHR^D26A^, and FSHR^F36A^ only via the cAMP pathway, whereas trans-activation of FSHR^L27A^ resulted in no cAMP but only activation of the IP signaling pathway. This form of trans-activation, subsequently named *intermolecular cooperation* to differentiate from the TMD-TMD trans-activation, maintains its specificity as the exo-FSHR could not trans-activate the LHR (36). Similar results were obtained by the group led by Jeoung (38).

Intermolecular cooperation was further tested in mice, using the *LHR* knockout mice (LuRKO), to avoid cofounding effects by the presence of the WT LHR, and signal-deficient and binding-deficient mutant LHRs. The expression of the two mutants was able to partly rescue LHR signaling, and generate measurable levels of testosterone, which was enough to rescue the fertility of male mice (40). Although, these experiments were performed using the LHR, one could speculate, due to their structural similarities and previous results *in vitro*, that the FSHR would be able to do the same. Evidence supporting intermolecular cooperation continuous to accumulate over the years (44, 45).

One lesson we can learn from intermolecular cooperation is that a ligand-exodomain is sometimes able to activate single pathways on the binding-deficient receptor, pointing out that GPCRs have different *switches* for triggering different pathways. This is a major focus in pharmacology where the search for compounds that trigger bias signaling is of clinical importance.

## FSHR Biased Signaling

**Figure 2:**
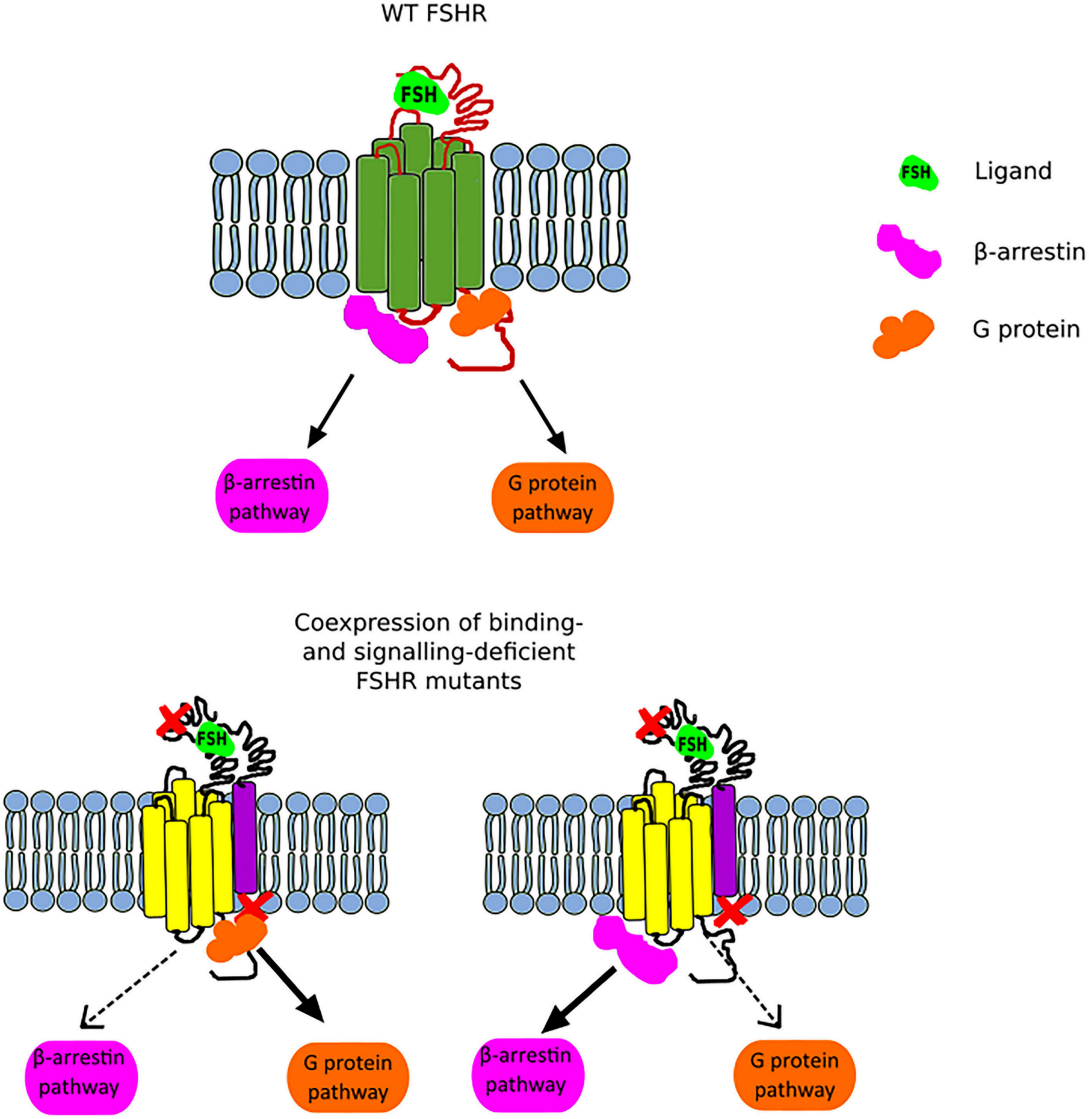
Scheme illustrating the FSHR biased signaling. The upper part of the figure presents the G protein-dependent and β-arrestin-dependent signal transduction of the wild-type FSHR. The lower part of the figure shows the bias toward either the G protein-dependent pathway **(left)** or the β-arrestin pathway **(right)** when a binding-deficient and a signal-deficient FSHR are co-expressed.

Another interesting issue related to trans-activation constitutes the phenomenon of *biased signaling*. Nevertheless, in order to be able to expound the biased signaling in the context of the FSHR, it is necessary to first clarify this term and take into account the entire GPCR family. GPCRs adopt multiple conformational states, both active and inactive. Each conformation is associated with different signaling pathway, and thus various downstream effects (46). Therefore, the presence of one particular conformational state can lead to the recruitment of G proteins and/or β-arrestins and thus lead to the activation of different signaling pathways (Figure 2). Furthermore, biased signaling may also be considered for the strength to which the signaling pathways are activated. Biased agonism occurs when binding of certain ligands to a GPCR results in transduction of different intracellular signaling to varying extents. Although most of the studies on GPCR biased signaling have been carried out on the β2AR and angiotensin receptors, it is speculated that this phenomenon concerns the vast majority of GPCR family (46, 47).

In the case of FSHR, we can distinguish three main types of biased signaling. The first type may occur due to the attachment of small chemical ligands to the FSHR transmembrane domain resulting in a stabilization of conformation and thus leading to biased signaling. This category of biased signaling arises greatest interest in the scientific community due to its therapeutic potential. The second type constitutes a consequence of modifications in the receptor structure due to the presence of mutation or polymorphism. In turn, the last type, also referred as *conditional effectiveness*, is caused by the presence of interacting proteins in the same environment as the FSHR (48). All these are of physiological and clinical importance as we will discuss below.

Clinical treatment using hormones is associated with the presence of a wide range of side effects resulting from the activation of multiple signaling pathways. Therefore, the possibility of influencing single signaling pathways constitutes another useful tool during research aimed at eliminating the side effects of hormone therapy (36).

With the above in mind, a high throughput screen for FSHR modulators that induce biased signaling was carried out by the group led by James A. Dias in collaboration with Addex Pharmaceuticals. They identified a small chemical molecule referred as ADX61623 which behaves as a negative allosteric modulator (NAM) of FSHR. When FSH and ADX61623 were simultaneously bound to the FSHR of rat granulosa primary cells, no cAMP nor progesterone production but concurrent estrogen production occurred. Nonetheless, *in vivo* studies carried out on female rats did not find any effect in follicular development (49). Three years later, the same research group described two new NAMs, structurally similar to ADX61623, termed ADX68692 and ADX68693. Whilst ADX68692 effectively inhibited cAMP, progesterone production and oestradiol synthesis in rat granulosa cells, as well as follicular growth in female rats, ADX68693 blocked cAMP and progesterone production but it was ineffectual in the case of oestradiol and *in vivo* experiments (50, 51).

In addition to studies focused on NAMs and their potential use in contraception, attention was also devoted to development of a positive allosteric modulators (PAMs) aimed for the treatment of infertility. Sriraman et al. discovered the efficacy of a thiazolidinone derivative as a PAM of FSHR. The treatment of rat granulosa cell line with aforementioned compound resulted in activation of ERK/Akt pathways and increased production of oestradiol. The latter was also present in the case of human granulosa cell line treated with this compound. Furthermore, stimulation of cultured preantral follicles with the thiazolidinone derivative triggered their maturation, in line with its FSH-like properties. Although, this compound has high genotoxicity and unfavorable pharmacokinetics and oral availability (52).

FSHR biased signaling was first identified in clinical samples by Aittomaki et al. in 1995 as the cause of hereditary hypergonadotropic ovarian failure in women and subfertility in men. A mutation at position 189, alanine to valine A189V, caused complete suppression of cAMP/PKA pathway, whereas it induced the ERK/MAPK pathway (53). Detailed *in vitro* experiments found that FSHR-A189V-induced ERK phosphorylation via β-arrestins (54), and that this was the result of the number of membrane-localized receptors—since an increase of receptor number in the presence of phosphodiesterase inhibitors resulted in elevated cAMP generation via G proteins. Thus, this suggests that the density of FSHRs affects the biased signaling where activation of G proteins requires a higher density of the FSHRs than those required by β-arrestins (55).

## FSHR Oligomerization

In the case of rhodopsin-like receptors, class A GPCRs, oligomerization is not a prerequisite for signaling occurrence, but it constitutes another form of its regulatory mechanism that can affect membrane expression of the receptor, ligand binding, desensitization as well as signal transduction (21).

FSHR oligomerization was first observed on the surface of Chinese hamster ovary using a confocal microscope in 1994 (56). However, for further biochemical and biophysical studies it was necessary to wait for the development of techniques and equipment. Nevertheless, before the presence of FSHR oligomers was confirmed, a number of studies have been carried out on the other two glycoprotein hormone receptors. Oligomers formed by the LHCGR were first reported in studies carried out by Osuga et al. (54) They co-expressed the binding-deficient LHCGR and either the chimeric receptor composed of FSHR extracellular region and LHCGR transmembrane domain or *N*-terminally truncated LHCGR, thereby providing information on the interactions between the two receptors (54). Shortly thereafter, the presence of TSHR dimers and oligomers was observed in thyroid tissue using FRET (57). Tao et al. revealed that LHCGR self-associates and forms both dimers and oligomers (58). Other research groups have shown that activation of LHCGR with either of its ligands causes clustering (59), whereas in the case of TSHR, stimulation with agonist results in cluster dissociation (60).

The crystal structure of FSHR revealed a dimeric complex composed of FSH-bound FSHR exodomain, or hormone-binding domain, (FSH/FSHR_HB_), suggesting that this dimer is, at least one of, the functional form of FSHR. The atomic organization also showed that FSHR oligomers are formed via hydrophobic interactions between LRR 2–4 (42). Next was to find FSHR oligomers in cells, which was firstly reported by the group of Richard M. Thomas, involving complementary coimmunoprecipitation of Myc- and FLAG-tagged FSHRs. This revealed the presence of FSHR oligomers in the ER, suggesting that the formation of FSHR oligomers takes place at the early stages of the biosynthesis of this receptor. Unlike the LHCGR and TSHR, the FSHR oligomers seem to be barely affected by ligand stimulation. An additional peculiarity of the FSHR, as compared to other glycoprotein receptors, is that its *C*-terminal is proteolytically processed just as oligomerization during protein biosynthesis—which was a limitation to tag this receptor using fluorescent proteins at its *C*-terminus (61). The constitutive formation of FSHR oligomers during the protein biosynthesis was confirmed by the group led by Rongbin Guan. They have also showed that the exodomain and the serpentine region are involved in the oligomerization (62). The issue of inserting a tag or florescent protein fusion to the *C*-terminus of FSHR was solved by Mazurkiewicz et al. by creating a construct in which the *C*-terminus of FSHR was replaced by that of the LHR, to which the fluorescent protein (FP) was subsequently joined to form the FSHR-LHRcT-FP chimera (63).

Another indication of FSHR homomerization, and one that suggests functional consequences, constitutes the phenomenon of negative cooperativity reported by a number of independent research groups. This allosteric mechanism involves decreased binding affinity of one homodimer-forming receptor due to the attachment of the ligand to the second protomer (64, 65). As a result of hormone-binding by FSHR, conformational changes in the receptor occur (65). As mentioned above, the existence of the FSHR oligomerization is also fully supported by the phenomenon of its trans-activation and intramolecular cooperation described, among others, by Ji et al. (36).

Interestingly, a few years after revealing the first crystal structure of the complex built of FSH and part of the FSHR exodomain, a novel crystal structure was unveiled. In contrast to the earlier model, this one includes the entire exodomain of FSHR (FSH-FSHR_ED_ complex) as well as a hinge region. This model revealed the presence of trimers in an asymmetrical unit (66) that can bind either one molecule of fully glycosylated FSH or three molecules of deglycosylated FSH (67).

### FSHR/LHCGR Heteromerization

**Figure 3:**
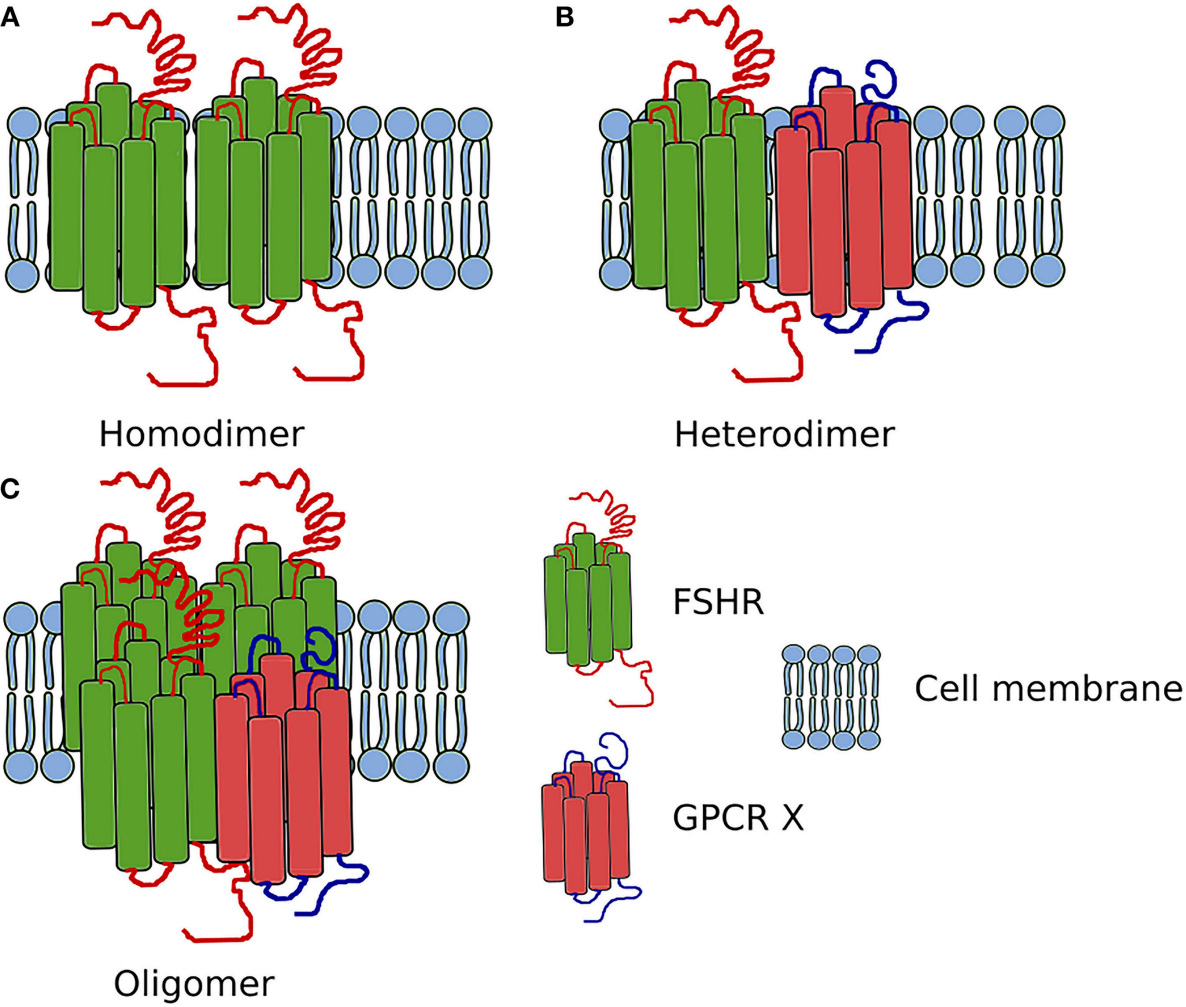
Scheme showing the FSHR complexes. **(A)** Homodimer composed of two FSHR protomers. **(B)** Heterodimer composed of one FSHR protomer and one different GPCR X protomer. **(C)** Oligomer composed of FSHR protomers and one different GPCR protomer (in ratio 3:1).

In addition to the self-association of FSHR, this receptor also heteromerizes (Figure 3). While in testes the FSHR and LHCGR are expressed in different cells types, in the ovary both receptors are expressed at least in one cell type—granulosa cells. Therefore, the heteromerization and cross-talk between these two receptors is of particular interest.

**Figure 4:**
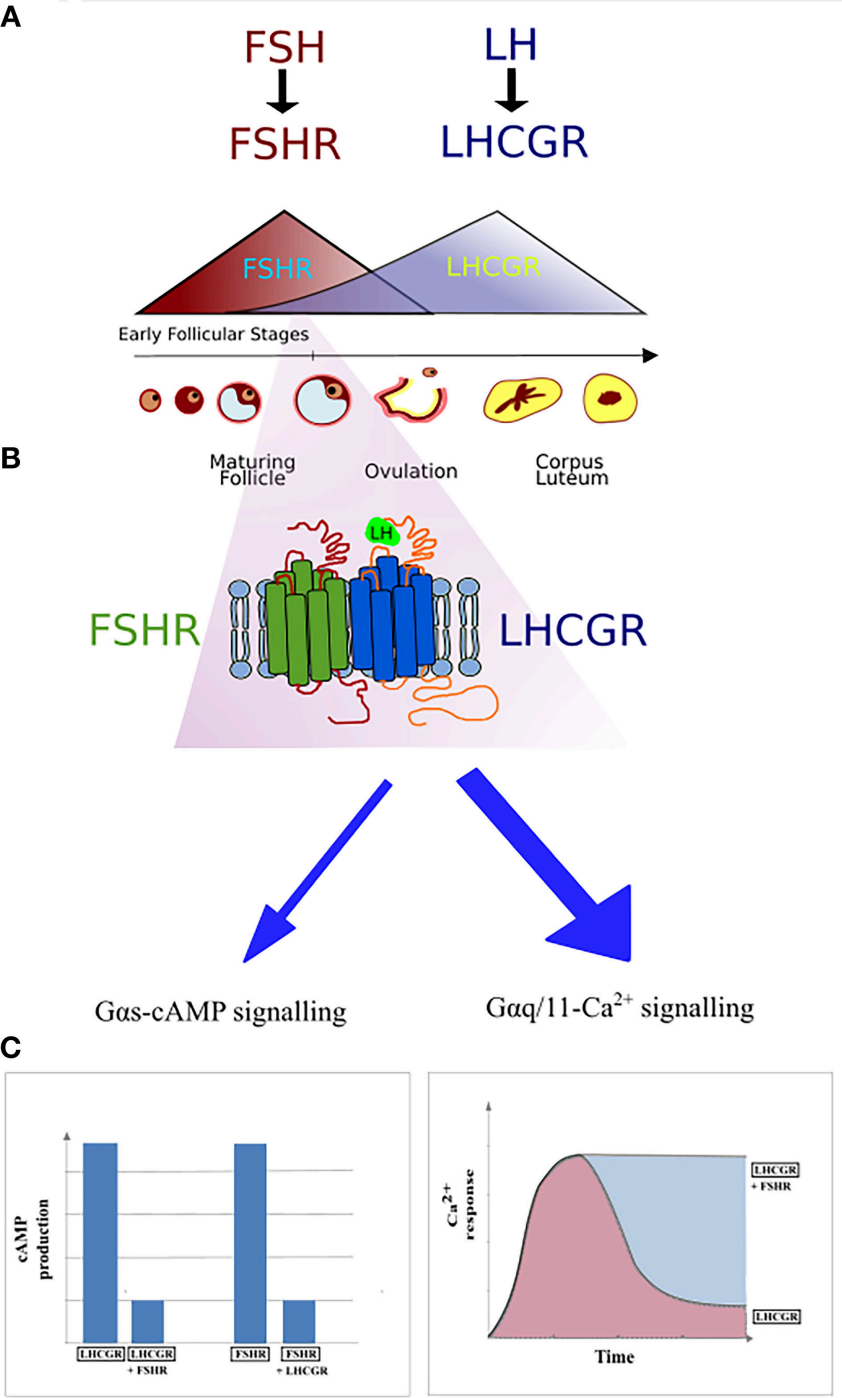
Schematic representation of FSHR and LHCGR expression in the ovarian granulosa cells during the menstrual cycle. **(A)** The domination of FSHR expression is noticeable during the first half of the cycle, while the second half of the cycle is dominated by the LHCGR expression. However, small expression of the LHCGR receptor is present during the early follicular stages. **(B)** During the simultaneous expression of LHCGR and FSHR both receptors form heterodimers. **(C)** Co-expression of liganded LHCGR and non-liganded FSHR results in either decreased cAMP production or prolonged Ca^2+^ release in LHCGR.

The first report on the heteromerization between the FSHR and LHCGR was made as a result of an experiment carried out by Feng et al. (65) in 2013. In that study, HEK293 cells were transiently transfected with either the hFSHR-Renilla luciferase (RLuc) and LHCGR-GFP pair or the LHCGR-RLuc and hFSHR-GFP pair. Importantly, the expression of the RLuc constructs was constant, whereas the expression the GFP^2^ constructs was incremented. Following co-transfection of HEK293 cells, BRET was performed showing that the FSHR and LHCGR form heteromers on the cell membrane. Interestingly, dimerization of LHCGR with FSHR resulted in the attenuation of the cAMP production upon stimulation with either hCG or LH, whereas FSH stimulation led to the attenuation of cAMP production (Figure 4). Therefore, the results of this study show that heteromerization between these two receptors occurs and that it leads to the attenuation of hormone-dependent signaling. This is physiologically relevant since these two receptors play essential roles in signal regulation in mature granulosa cells in ovaries (68).

The work of Mazurkiewicz et al. provided another evidence of FSHR/LHCGR heteromerization. HEK293 cells co-transfected with a pair of constructs—hFSHR-rLHR-cT-mCherry and LHR-YFP, revealed the presence of FSHR/LHCGR heteromers on the plasma membrane by FRET, thereby confirming previous reports (63).

More detailed reports on FSHR/LHCGR heteromerization suggest that LHCGR couples with the G_α*q*/11_ protein that activates phospholipase C. That in turn lead to increased concentration of intracellular diacyl glycerol and inositol phosphates triggering the release of Ca^2+^. Nonetheless, the G_α*q*/11_-Ca^2+^ signaling is induced only under conditions of high LHCGR membrane expression and high hormone concentrations. HEK293 transiently transfected with either HA-FSHR or FLAG-LHCGR or both plasmids simultaneously, established that only co-expression of both receptors simultaneously induced a sustained LH-induced Ca^2+^ release, showing that the non-liganded FSHR is able to disrupt the G_α*q*/11_-dependent Ca^2+^ signaling of engaged LHCGR. Primary human granulosa cell line expressing both receptors resulted in similar results to those derived from the experiment with HEK293 cells. The LHCGR/FSHR crosstalk is able to alter LHCGR signaling, presumably constituting a regulatory mechanism of the functions of granulosa cells (69).

The phenomenon of FSHR/LHCGR heteromerization seems to play an important role in terms of antral follicular development. A recently suggested model infers that low expression of LHCGR is yet noticeable during the FSH/FSHR-dependent early antral stages (Figure 4). At the time, both receptors form heteromers, and binding of FSH to its cognate receptor results in the trans-activation of LHCGR in an environment of very low LH. Low LH is likely required for keeping LHCGR-expressing theca cells under low steroidogenesis while proper follicular selection occurs. Therefore, FSHR/LHCGR crosstalk in granulosa cells may explain their steroidogenic activity at this stage of folliculogenesis, an activity otherwise associated to LH-activation in large antral stages (70). Clinical and *in vivo* studies will help to elucidate some of the remaining unsolved conundrums in folliculogenesis and its relation with FSHR/LHCGR interactions.

### Methods to Investigate the Oligomerization of FSHR

Oligomerization and trans-activation of GPCRs have taken long to be widely accepted, due to (partly) the need for novel techniques and equipment such as super-resolution microscopy, to visualize these molecules with enough definition. One of the first and most common methods used to study GPCR oligomerization was co-immunoprecipitation (co-IP) (64). This is a technique that allows the precipitation of protein complexes using specific binding antibodies, followed by visualization with SDS-PAGE and Western Blotting. The advantages of co-IP include relatively low costs and its simplicity. The key issue for the widespread use of co-IP for detection of GPCRs homo- and heterodimers was the cloning of epitope-tags on GPCRs (71). However, co-IP requires many controls to rule out non-specific aggregation, non-specific antibody binding and appropriate lysis protocols. An additional limitation of this method is the lack of specific antibodies recognizing endogenous GPCRs (64, 72, 73). Co-IP has been used to detect many oligomeric complexes, including Myc- and FLAG-tagged FSHRs. Discovering that FSHR forms oligomers at the early stages of biosynthesis as mentioned above (61).

Another biochemical method allowing the electrophoretic separation of intact protein complexes is Blue Native Electrophoresis (BN-PAGE). This method is characterized by the use of non-denaturing detergents that do not affect the quaternary structure of proteins, unlike SDS. In addition, Coomassie Blue dye gives the proteins a negative charge and prevents non-specific protein aggregation during electrophoresis. Visualization of the examined types of GPCR is possible by means of immunoblotting (74, 75). The use of this method allows simple determination of the proportion of different oligomeric types of GPCR (76).

Förster resonance energy transfer (FRET) is a method for studying the interaction between proteins in a living cell. The phenomenon of resonance energy transfer between donor and acceptor was pioneered by Förster (77). Non-radiative energy transfer occurs between one excited fluorescent protein and another at a distance no >100 Å. An additional condition for the occurrence of this phenomenon is the overlap of the emission spectrum of the donor molecule and the excitation spectrum of the acceptor molecule. The most frequent donor-acceptor pair is GFP and one of its variant with different spectral characteristics (78, 79). Using FRET it is possible to detect both ligand-induced signal transduction of GPCR (80), activation of GPCR in response to ligands (81) and GPCR oligomerization (82). In FRET studies two different GFP variants, meeting the energy transfer conditions, are linked to the same or different type of GPCRs or GPCR and their corresponding ligands. Detection of the ratio of donor:acceptor protein emission spectrum indicates the interaction between the proteins tested. Nonetheless, the fluorescent proteins used in this method are relatively large, which can affect the interaction or function of the proteins (83). In addition, accuracy of measurements can be affected by bleed-through, background signals, cross-talk and photobleaching (83, 84). This method has been used repeatedly for studies on oligomerization of the full-length native FSHR. The use of specific monoclonal antibodies or Fab fragments that have been tagged with various fluorophores has shown that FSHRs are present as oligomers on the plasma membrane (61).

Some of the limitations of FRET have been solved by Time Resolved FRET (TR-FRET), which is based on the use of a fluorescent donor containing lanthanides. Terbium and europium are characterized by long-lasting fluorescent light emission. This results in a significant reduction in noise, and thus a higher signal-to-noise ratio. These elements are trapped in stable cryptates that absorb light and transfer energy to the lanthanide (85, 86). Receptors can be tagged non-covalently using antibodies or covalently using tag proteins such as SNAP, CLIP or Halotag. If the labeled receptors form dimers, the tags are located close to each other and a TR-FRET signal is generated (87).

Bioluminescence resonance energy transfer (BRET) is the transfer of energy from a luminescent donor to a fluorescent protein as an acceptor. Depending on the type of substrate, the luciferase and the fluorescent protein, the BRET method can be divided into: BRET1, BRET2, BRET3, eBRET, and QD-BRET (88). In first generation of BRET (BRET1), the energy transfer pairing was the most popular bioluminescent protein—RLuc and enhanced yellow fluorescent protein (eYFP). The substrate of RLuc is coelenterazine (71, 89). Nevertheless, the BRET1 system is distinguished by the presence of a high background due to the bleed-through of donor emission peak at the acceptor emission wavelength. Therefore, the second generation of BRET (BRET2) was developed with a better separation of the acceptor and donor spectra in comparison to BRET1. This was achieved by the use of different substrate, referred as DeepBlueC, which is an analog of coelenterazine that is characterized by lower emission interference with the acceptor, better biophysical properties, cell permeability as well as reduced toxicity. DeepBlueC is used with modified GPF2 instead of eYFP. Nonetheless, DeepBlueC has some disadvantages that include its short lifetime and low light emission. In the third generation of BRET (BRET3) the firefly luciferase is used as a donor, while various types of fluorescent proteins that are excited by the wavelength emitted by luciferase (565 nm) are used as acceptors. Other types of BRET constitute extended BRET (eBRET) that enables to monitor experiments in real-time using coelentrazine as well as QD-BRET based on the use of quantum dots (90, 91). Compared with FRET, the advantage in favor of this method is that there is no need to use a light source to excite the donor and thus no-photobleaching. However, BRET is characterized by low sensitivity and to be unable to determine the location of the signal in a cell (71). BRET is a method enabling the study of oligomerization (92) and GPCR interactions with other proteins as well as receptor signaling and activation (90).

Determining the structure GPCR is performed by X-Ray crystallography. This method can contribute to the increase of our knowledge about the ligand-receptor and receptor-effector protein interactions, as well as GPCR oligomerization. However, care should be taken as some of these interfaces might be artificial and potentially may not represent a functional biological assembly (91).

Beside the methods to detect protein-protein interaction, the raise of biosensors and assays to analyze secondary metabolites and signaling players have been vital to determine the functionality of oligomers and complementing receptors (91, 93–95).

## Conclusions

The last several years of research centered around the FSHR shed new light on the processes of its biosynthesis, maturation, membrane expression, activation and intracellular signaling. In this review we have discussed the evidence on the existence of trans-activation, oligomerization and biased signaling of the FSHR. Oligomerization studies revealed that the FSHR is present not only as a monomer, but it also forms higher-order complexes such as homo- and heteromers (with the LHCGR). Homomerization occurring during the protein biosynthesis constitutes the quality control checkpoint at the ER level. The discovery of FSHR oligomers resulted in further dissecting the interaction between receptors, which led to the conclusion that the FSHR can be activated not only via cis- but also trans-activation. Furthermore, trans-activation studies have also provided evidence for the existence of biased FSHR signaling at various levels. All aforementioned phenomena constitute FSHR regulatory mechanisms of intracellular signaling control through the determination, modification and fine-tuning of the signal.

Better insight in the molecular mechanisms and functioning of the FSHR may contribute to the development of new drugs to trigger single signaling pathways. Furthermore, it is worth noting that the importance of FSHR/LHCGR heteromerization is mainly due to their co-expression on the surface of granulosa cells and the participation of both receptors in the ovulation process. The expression of FSHR and LHCGR on the plasma membrane as well as the level of gonadotropins fluctuate during the menstrual cycle—the FSH/FSHR prevails during the first phase of the cycle, whereas the second phase is dominated by the LH/LHCGR. The expression of FSHR is induced at the beginning of the menstrual cycle and then decreases with the maturation of the ovarian follicle. Afterwards, the expression of *LHCGR* is induced due to the FSH-stimulation, via the FSHR, plus other factors. Following this, the LHCGR expression decreases upon desensitization by LH surge (Figure 4) (96). It is believed that the crosstalk between both receptors is responsible for switching the dominance between the FSHR and LHCGR expression during the cycle as a result of affecting the LHCGR and LH signaling (69). Therefore, disturbed interactions between FSHR and LHCGR may cause the development of several diseases. Presumably, crosstalk disturbances may lead to the overexpression of LHCGR and thus to the development of polycystic ovary syndrome (97, 98).

## Author Contributions

KS and AR-M outlined the manuscript. KS, JK, AP, BP, and MK collected the information and co-wrote the manuscript. GA and KS made the graphical work. AR-M coordinated the work and supervised the accuracy of the information. All authors critically read and approved the manuscript.

### Conflict of Interest Statement

The authors declare that the research was conducted in the absence of any commercial or financial relationships that could be construed as a potential conflict of interest.
